# Development of mutome-specific personalized vaccines for pancreatic cancer

**DOI:** 10.1186/2051-1426-1-S1-P224

**Published:** 2013-11-07

**Authors:** Eric Lutz, Heather Kinkead, Allison Yager, Todd Armstrong, Peter Lauer, Dirk Brockstedt, Thomas Dubensky, Elizabeth Jaffee

**Affiliations:** 1 Oncology, Johns Hopkins University, Baltimore, MD, USA; 2 Aduro Biotech, Berkeley, CA, USA

## 

A major obstacle to the development of immunotherapy for pancreatic ductal adenocarcinoma (PDA) has been identifying immune relevant antigens that are not expressed by normal cells. DNA sequencing technologies that allow for the genome-wide identification of tumor mutations have shown that human PDAs harbor an average of 63 mutations (called the “mutome”). Since these mutations are not present prior to the onset of tumorigenesis, each mutation generates a novel and essentially foreign antigen (termed “epitope”). Unlike most classical tumor epitopes, mutome-derived epitopes are uniquely specific to each patient’s tumor. Furthermore, T cells recognizing them may avoid immunological tolerance mechanisms that inhibit or deplete T cells that recognize classical tumor epitopes that are also expressed by normal cells. Vaccines designed to activate mutome-specific T cells have the potential to induce robust and specific antitumor responses and provide a new approach for personalized tumor immunotherapy. We are using the Panc02 murine PDA model to develop and explore the potential of this approach. Sequence analysis identified 596 point mutations in Panc02 cells, significantly more than the number found in human PDAs. The NetMHC prediction server was used to screen for epitopes predicted to bind H2-Db and H2-Kb. In total, 15 strong candidates (NetMHC scores <50) and another 85 candidate epitopes within the limits of prediction (scores <500) were predicted. We analyzed the *in vivo* immunogenicity of 81 mutations predicted to generate epitopes with scores <1000 by immunizing mice with 20mers spanning each mutation with Poly (I:C) and addavax as adjuvants. IFNγ ELISPOT analysis demonstrated that 14 of the 81 (17.3%) mutations generated immunogenic CD8+ T cell epitopes, and 5 of the 14 induced robust IFNγ responses (>100 spots per 1x10^5^ T cells). Antitumor efficacy was evaluated by immunizing mice 3 days following subcutaneous Panc02 challenge. Only immunization against the 5 robust peptides was capable of preventing tumor outgrowth, and was significantly more effective than treatment with GM-CSF-secreting whole-cell vaccination (GVAX), another approach currently being investigated in the clinic by our group. We are currently testing the efficacy of this approach against larger established tumors and metastatic liver lesions. We are also working to optimize this approach by testing additional adjuvants and vaccine vectors, including recombinant *Listeria monocytogenes* that express the mutant gene sequences. Exact epitopes are being mapped in order to evaluate the utility of MHC-binding predictions. Ultimately, we intend to translate our optimized approach into the clinic.

